# Atypical Osteomyelitis Caused by *Mycobacterium chelonae*—A Multimodal Imaging Approach

**DOI:** 10.1155/2013/528795

**Published:** 2013-03-31

**Authors:** Roland Talanow, Hendryk Vieweg, Reimer Andresen

**Affiliations:** ^1^EduRad, Lincoln, CA, USA; ^2^Institute of Diagnostic and Interventional Radiology/Neuroradiology, Westküstenklinikum Heide, Academic Teaching Hospital of the Universities of Kiel, Luebeck and Hamburg, Germany

## Abstract

We present an unusual case of a biopsy-proven *Mycobacterium chelonae* infection (MCI) of skin and soft tissue, which led to osteomyelitis in a 55-year-old Caucasian male. We provide clinical data and discussion about MCI and its diagnostic workup and demonstrate comprehensive imaging findings, including clinical pictures, radiographs, three-phase bone scintigraphy, and combined SPECT/CT findings of this entity, which have not yet been presented in the medical literature.

## 1. Introduction

*Mycobacterium chelonae* is an atypical mycobacterium which does not cause tuberculosis or leprosy in humans. They are known for infections of skin, lungs, eyes, and soft tissue. People with immunodeficiency are more likely to contract an infection although a minority of concerned patients were reported to be primary healthy. The identification of the agent is performed by bacterial cultures, polymerase chain reaction (PCR), and DNA sequencing [1].

Bone and joint infections are extremely rare in the context of atypical mycobacterium infections [2].

Osteomyelitis is mostly caused by staphylococcus aureus (75–80%), infrequently also by other bacteria, viruses, and funguses [2, 3].

In the medical literature we found only 2 cases of *Mycobacterium chelonae* osteomyelitis visualized by multiple diagnostic imaging modalities [2, 4]. To our best knowledge SPECT/CT findings have not yet been published at all.

## 2. Clinical Presentation

The patient is a 55-year-old Caucasian male with an unclear redness and swelling at the right medial malleolus without any noticed trauma. His medical record includes ulcerative colitis and sarcoidosis treated with prolonged cortisone therapy and liver transplant for treatment of primary sclerosing cholangitis (PSC) 14 years ago.

**Figure 1:**
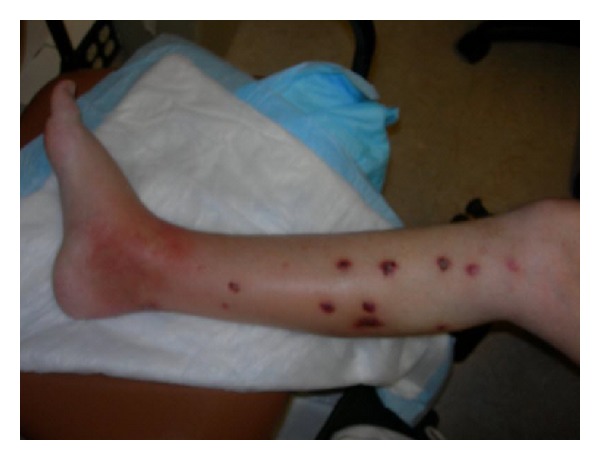
Clinical picture shows swelling and erythema over the medial aspect of the right ankle. In addition nodular lesions on the lower leg are seen.

Tentative diagnosis was gout because of high uric acid in blood testing but common ambulant drug therapy with colchicine and indomethacin did not improve the condition. Spreading of the swelling and redness in combination with increasing inflammation parameters was interpreted as possible phlegmon and treated with penicillin. However no regression was observed and the patient developed additional nodular lesions spreading from the ankle to the anterior medial lower leg. Visible were 12–15 nodular lesions, the newest and proximal lesions were erythematous, and the older ones were darker and violaceous with some scaling (Figure 1).

In the following, punch biopsies were performed, which microscopically showed positive results on gram staining and acid resistance on Ziehl-Neelsen staining, matching with mycobacteria. Bacteria cultures revealed rapidly growing mycobacteria of Runyon group IV.

Additional PCR and sequencing of the amplified DNA detected *Mycobacterium chelonae*.

In the course of the events several diagnostic imaging modalities were conducted to find out about a possible osseous involvement, which is important for the appropriate selection of antibiotics.

**Figure 2:**
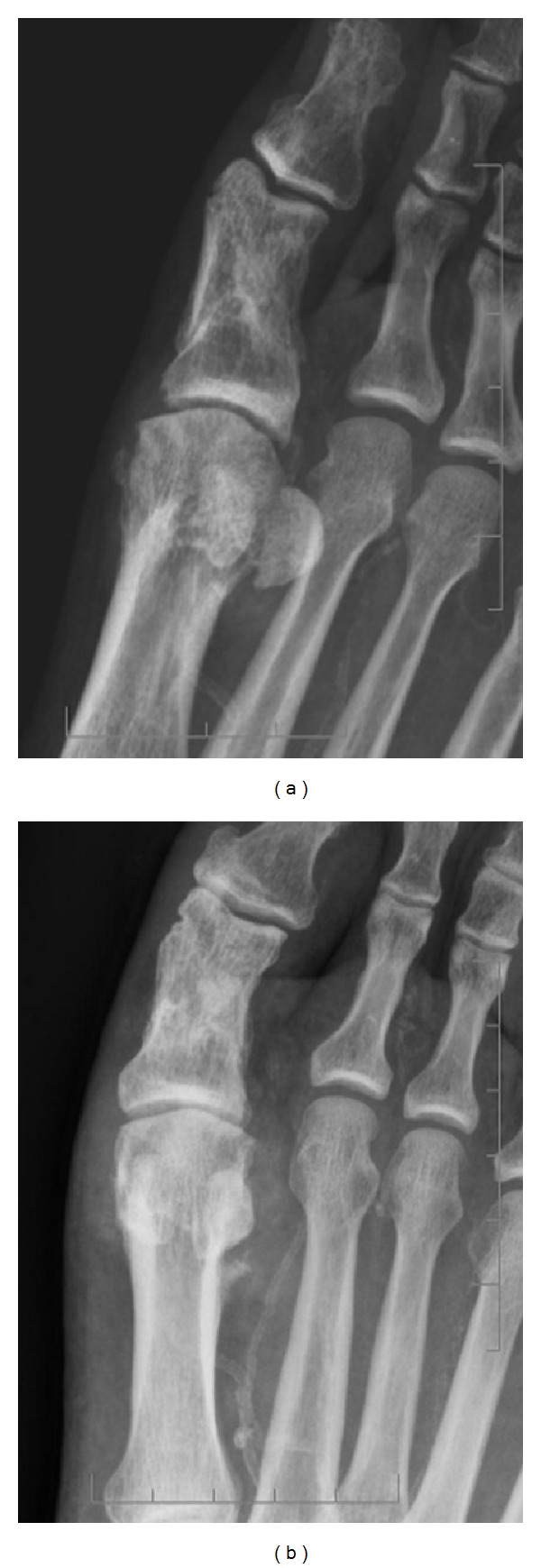
Initial radiographs show focal soft tissue reaction around the first MTP joint (emphasized by softer image windowing on (b)) without signs of bone affection. Healed fracture of the proximal first phalanx.

Initially radiographs of the right ankle, tibia, and fibula were obtained, showing moderate circumferential soft tissue swelling around the metatarsophalangeal joint I (MTP I). The ankle mortise was intact with the underlying bony structures appearing normal, beside a healed fracture at the proximal first phalanx (Figure 2).

For further investigation MRI was scheduled but even with sedation refused by the patient due to claustrophobia.

**Figure 3:**
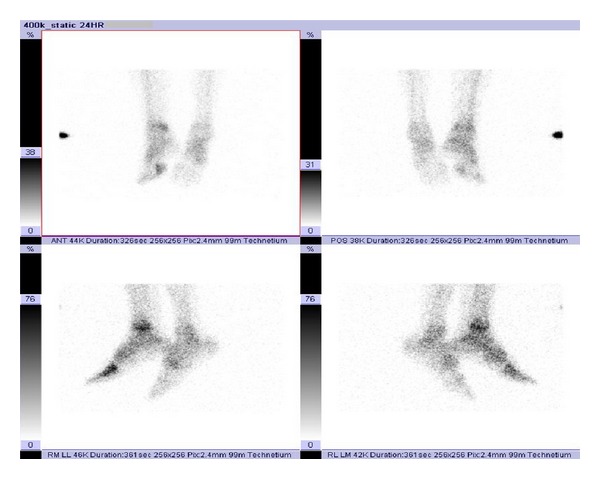
Three-phase bone scan was performed, with attention to the ankles and feet (33 mCi Tc-99 m MDP injected IV). The images demonstrate areas of increased delivery of tracer at the right distal lower extremity and around MTP I on bone phase.

Instead a three-phase bone scan (33 mCi Tc-99 m MDP IV with 24-hour delay) was performed which showed local hyperemia at the right distal lower leg and the MTP I on perfusion phase, surrounding soft tissue tracer uptake on blood pool phase and increased bone remodeling on bone phase, also at the right distal lower leg and the MTP I (Figure 3, bone phase).

Subsequently, using the already given tracer, a combined SPECT/CT was conducted which provides the advantages of better localization and demonstration of morphological changes, compared with scintigraphy. 

**Figure 4:**
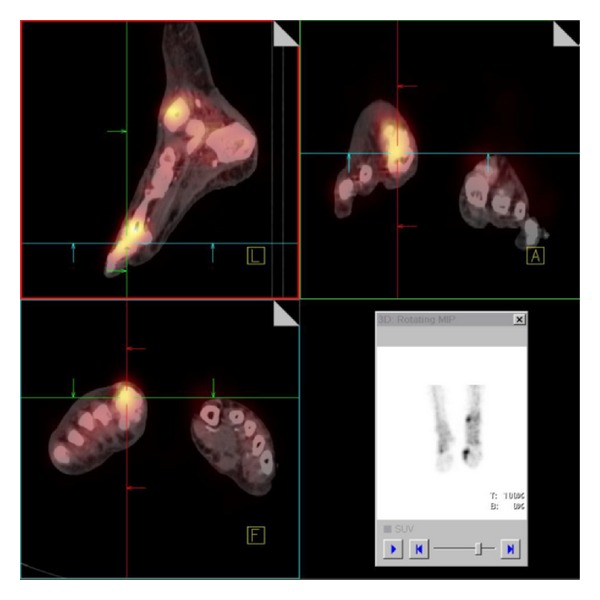
Merged SPECT/CT imaging of the lower extremities localizes the increased tracer uptake exactly at the distal tibia and MTP I.

**Figure 5:**
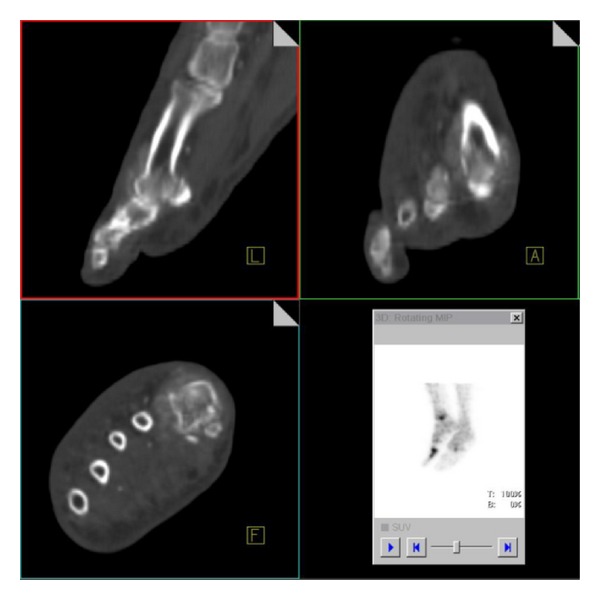
CT-data of the combined SPECT/CT demonstrates soft tissue and periosteal reaction and mild osseous disruptions at the MTP I.

In the merged pictures we could localize the increased tracer uptake exactly at the distal tibia and the MTP I (Figure 4). The CT data showed evidence of soft tissue and periosteal reaction and osseous disruption at the MTP I (Figure 5). 

The combination of findings was indicative for a soft tissue infection with additional multifocal osteomyelitis, at the MTP I already with initial osseous destructions.

Antibiotic therapy was adjusted, following antibiogram, to a combination of azithromycin, moxifloxacin, and meropenem under which the patient improved. He could be discharged from our hospital in a good condition 2 weeks later.

## 3. Discussion

Mycobacteria are a heterogeneous group of aerobic bacteria. There are significant differences in the appropriate treatment so that it is important to distinguish between the agents.

The group can be detected by being gram positive and acid-fast on gram and Ziehl-Neelsen staining. The Runyon classification divides into 4 groups, based on growth rates in bacterial cultures. PCR and DNA sequencing are helpful to detect the agent.

The most familiar mycobacteria are *Mycobacterium tuberculosis* and *Mycobacterium leprae*, both listed in Runyon Group III.

*Mycobacterium chelonae* is a rapidly growing species (Runyon group IV) of mycobacteria other than tuberculosis (MOTT). Those do not respond to first-line antituberculosis drugs. 

It is found in natural and processed water sources and known to be responsible for a distinct number of primary infections of the skin, lung, eyes, and soft tissue [2, 5–7]. 

As an opportunistic pathogen it should be considered when unclear skin and soft tissue infections occur on immunosuppressed patients like patients on corticosteroid therapy, after organ transplantation and with immunodeficiency or autoimmune disorders. 

Infections of the bone and joints by nontuberculous mycobacteria, such as *Mycobacterium chelonae*, are extremely rare and often overlooked with less than a dozen reported cases in the medical literature [2, 8]. However for the selection of antibiotic therapy and for avoidance of chronification, it is of high priority to be aware of possible bone affection. 

The disease is often a disseminated cutaneous infection [9, 10], like in our case. If osteomyelitis takes place in this regard, it is usually localized [9, 11] with only one reported case of multifocal osseous involvement [2]. Our findings are indicative for bifocal affection of the distal tibia and the MTP I. 

Osteomyelitis by *Mycobacterium chelonae* has occurred occasionally in the sternum after cardiac operations [12, 13] or in immunocompromised patients [8, 10, 14, 15]; however recent publications reported also occurrence in previously healthy patients [4, 6]. Sternal osteomyelitis is associated with a poor clinical prognosis, perhaps related to late diagnosis and consecutive late onset of specific treatment [12].

To our best knowledge only 2 manuscripts have been published, describing *Mycobacterium chelonae* osteomyelitis on imaging [2, 4]. So far no literature has been published, describing SPECT/CT imaging findings of osteomyelitis caused by atypical mycobacteria, especially by *Mycobacterium chelonae*.

All previous reports demonstrated advanced bone changes on imaging, already visible on X-ray. In our case, the initial radiograph was unremarkable for osteomyelitis but we were able to detect the infection with MDP bone scan and get information about localization and morphological changes with a combined SPECT/CT, facilitating appropriate antibiotic therapy.
